# Retinoblastoma mutation predicts poor outcomes in advanced non small cell lung cancer

**DOI:** 10.1002/cam4.2023

**Published:** 2019-02-17

**Authors:** Priyanka Bhateja, Michelle Chiu, Gary Wildey, Mary Beth Lipka, Pingfu Fu, Michael Chiu Lee Yang, Fatemeh Ardeshir‐Larijani, Neelesh Sharma, Afshin Dowlati

**Affiliations:** ^1^ Department of Hematology and Oncology Case Western Reserve University University Hospitals Seidman Cancer Center Cleveland Ohio; ^2^ School of Medicine Case Western Reserve University Cleveland Ohio; ^3^ Department of Hematology and Oncology University Hospitals Seidman Cancer Center Cleveland Ohio; ^4^ Department of Population and Quantitative Health Sciences Case Western Reserve University Cleveland Ohio; ^5^ Department of Pathology University Hospitals Cleveland Medical Center Cleveland Ohio; ^6^ Department of Internal Medicine University Hospitals Cleveland Medical Center Cleveland Ohio; ^7^ Department of Biomedical Research Novartis Pharmaceuticals Corporation East Hanover New Jersey

**Keywords:** genomics, immunotherapy, non small cell lung cancer, response, retinoblastoma, small cell lung cancer

## Abstract

The retinoblastoma gene (*RB1*) encodes the retinoblastoma (RB) pocket protein that plays an important role in cell cycle progression. Here we determine the frequency and prognostic significance of *RB1* mutation in non small cell lung cancer (NSCLC), restricting inclusion to Stage III and IV patients with linked genomic and clinical data. The primary outcome was median overall survival (OS). We identified *RB1* mutation in 8.2% of NSCLC patients. The median OS for wild‐type (wt) *RB1* was 28.3 months vs 8.3 months for mutant *RB1* (Hazard Ratio = 2.59, *P *=* *0.002). Of special interest, *RB1* mutation also correlated with lack of response to immunotherapy. Our study focused on *RB1* mutation in locally advanced and advanced non small cell lung cancer to better facilitate comparisons with small cell lung cancer (SCLC). In our SCLC cohort, *RB1* mutation was identified in 75% of patients and wt *RB1* was associated with significantly shorter OS (*P *=* *0.002). The different outcomes of *RB1* mutation observed among lung cancer subtypes suggest a more complicated mechanism than simple regulation of cell cycle or response to chemotherapy.

## 1 INTRODUCTION

Genomic sequencing of tumor DNA has changed the therapeutic landscape for non squamous, non small cell lung cancer (NSCLC) with the discovery of recurrent oncogenic driver mutations in *EGFR*,* ALK,* and *ROS‐1* that can be specifically and effectively targeted by new drugs. Additional targetable oncogenic driver mutations in *BRAF, NTRK1, HER2, RET,* and *MET* are found at lower frequency in lung adenocarcinoma patients.^1^ Although *KRAS* mutation is found in ~25‐30% of lung adenocarcinoma patients and remains largely untargetable,^2^ there is a suggestion these patients demonstrate favorable responses to immunotherapy, although co‐mutation in the tumor suppressor gene *STK11/LKB1* identifies a subset of *KRAS* mutant patients that show poor response to immunotherapy.^3^ Alteration in the tumor suppressor *NF1* was also suggested as a potential druggable target in NSCLC.^4^ Ultimately, about 30%‐40% of adenocarcinoma lacks a clearly identifiable oncogenic alteration.^5^

Genomic studies in small cell lung cancer (SCLC) have also identified subgroups with *MYC* amplification, *SOX‐2* amplification, *FGFR‐1* amplification, *PTEN* loss, *RICTOR* amplification, and *NOTCH* inactivation.^6^, ^7^ Genomic identification in SCLC has clearly lagged behind that of NSCLC in part due to tissue availability. Our group previously published on the genomics of small cell lung cancer and identified retinoblastoma (*RB1*) gene mutation status through targeted exome sequencing as a predictor of outcomes.^8^

Retinoblastoma was the first tumor suppressor gene to be discovered based on an association with a rare childhood tumor, retinoblastoma, that occurs at a frequency of 1 in 20 000 live births.^9^ The Knudson hypothesis of a second hit in retinal cells of children with germline mutation led to the understanding of how tumor suppressor genes drive the development of cancer. About 40% of retinoblastomas are hereditary and hereditary retinoblastoma survivors are at risk for second malignancies like osteosarcoma, melanoma, and epithelial malignancies like lung, bladder, and breast cancer.^10^, ^11^ Somatic alterations in *RB1* are known to occur in various malignancies including lung, breast, bladder, and prostate cancer.

Retinoblastoma encodes the retinoblastoma pocket protein (RB) that regulates the cell cycle by binding to E2F transcription factors in its unphosphorylated form to repress their activity. In response to mitogenic stimuli, the cyclin dependent kinases (CDK) phosphorylate RB, causing release of the binding to E2F and progression through the cell cycle. p16^INK4A^ and other CDK inhibitors maintain RB in the unphosphorylated, active form. The role of *RB1* is most understood in the regulation of G1 to S transition and cell proliferation. There are other roles attributed to RB like regulation of epithelial to mesenchymal transition^12^, ^13^ and a possible role in immune response.^14^ Here, we explore the association of *RB1* mutation status to outcome in advanced NSCLC. Our study focused on locally advanced and advanced NSCLC to better facilitate comparisons with SCLC, a disease with a defined role for *RB1*.

## 2 PATIENTS AND METHODS

We have an IRB approved institutional database that includes all lung cancer patients diagnosed at our institution and referred for thoracic oncology opinion. Patients were staged according to TNM7 staging (American Joint Committee on Cancer staging manual, 7th edition). All patients are discussed at our multidisciplinary tumor board and a TNM staging is assigned after the multidisciplinary discussion.

The inclusion criteria were locally advanced or metastatic (stage III and stage IV or recurrent NSCLC) disease, age greater than 18, seen at our institution from 2013 to 2016. We used the Foundation One sequencing platform which interrogates 315 genes exomes as well as introns of 28 genes involved in rearrangements (Supporting Information, Table S1).^15^ Patients who did not have next generation sequencing of tumor DNA were excluded. We collected data on age, sex, race, smoking status, stage, histological subtype for NSCLC, treatment with systemic chemotherapy, immunotherapy, and mutations. Smoking status was defined as yes for current smoker (who is smoking at the time of diagnosis or quit within 12 months of diagnosis) or former smoker (who quit at 12 months prior to diagnosis). Never smoker was defined as someone who smoked <100 cigarettes over their lifetime. The date of diagnosis is the date of biopsy and pathological confirmation of disease. The overall survival (OS) was calculated from the date of diagnosis to date of death and censored at the date of last follow‐up for survivors. Survivor distribution was estimated using Kaplan‐Meier methods and the difference of OS between the groups was examined by a log‐rank test. The effect of continuous measurements, including age, on OS was estimated using the Cox model. The effect of *RB1* mutation status in our NSCLC cohort on OS was further evaluated using the multivariable Cox model controlling for the effects of age, sex, stage, smoking, and chemotherapy. All tests are two‐sided and *P* ≤ 0.05 was considered statistically significant. The specific type of *RB1* mutation was not considered for outcome analysis, just its presence or absence. The characteristics of our SCLC cohort have been previously described.^8^, ^16^

The mutation distribution along the RB protein was plotted using cBioPortal mutation mapper. Immunohistochemistry (IHC) was performed on formalin‐fixed paraffin‐embedded (FFPE) specimens to evaluate RB expression using Cell Signaling‐ use capital Signaling Technology 4H1 mouse antibody (catalog number 9309). p16^INK4A^ IHC was done using the CINtec histology kit. p16^INK4A^ expression has been proposed as a surrogate for loss of RB protein expression or dysfunctional protein.^17^, ^18^, ^19^ IHC scoring was done by a thoracic pathologist. The intensity of IHC staining was graded as absent (0), weak (1+) or strong (2+) and focused on nuclear staining for RB and cytoplasmic staining for p16. In addition, the percent of tumor cells showing staining was scored separately.

## 3 RESULTS

One hundred and ninety‐five patients met the inclusion criteria for NSCLC and had available both clinical and genomic data. The mutation frequency of *RB1* in our cohort of NSCLC was 8.2%, which is consistent with prior reports and the TCGA database.^5^ The baseline characteristics (Table 1) of *RB1* mutant compared to *RB1* wt patients were well balanced between the 2 groups, except for a higher number of stage 3 patients in the *RB1* mutant NSCLC group.

**Table 1:** Baseline characteristics of NSCLC cohort

Factor	*RB1*: wild type *N* (%)	*RB1*: mutant *N* (%)	*P*‐value
Stage			
III	38 (21%)	7 (43%)	0.044
IV	139 (79%)	9 (56%)	
Smoking			
No	25 (14%)	0 (0%)	0.108
Yes	153 (86%)	16 (100%)	
Age			
Median (Range)	64 (33‐92)	59 (45‐85)	0.913
Race/ethnicity			
White	121 (72%)	7 (43%)	0.075
Black	43 (25%)	9 (56%)	
Asian	3 (2%)	0	
Hispanic	1	0	
Sex			
Male	102 (57%)	8 (50%)	0.589
Female	77 (43%)	8 (50%)	
Histology			
Adenocarcinoma	156 (90%)	12 (75%)	0.119
Squamous	10 (6%)	3 (19%)	
Large cell	3 (1%)	1 (6%)	
Adenosquamous	5 (3%)	0	

**Figure 1:**
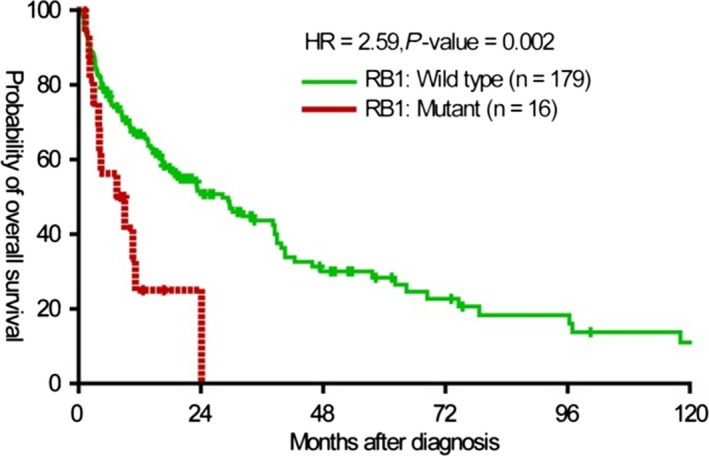
Kaplan‐Meier Curve for OS in NSCLC. *RB1* mutation was identified in 8.2% of NSCLC patients (16 of 195 patients). With a median follow‐up of 15.1 months, the median OS for wt *RB1* was 28.3 months and for mutant *RB1* was 8.3 months

In NSCLC, *RB1* mutant status when compared to *RB1* wt was associated with worse OS (8.3 months vs 28.3 months, Hazard Ratio (HR) = 2.59, 95% Confidence Interval (CI) = 1.4‐4.79, *P *=* *0.002) and this was statistically significant (Figure 1). On multivariate analysis (Table 2), after adjusting for age, sex, stage, smoking status, receipt of chemotherapy, and other gene mutations, *RB1* mutant status was still predictive of worse outcomes in NSCLC (HR = 3.07, 95% CI = 1.54‐6.14, *P *=* *0.002). While the mutation status of seven other genes also predicted worse outcomes, only *MLL2* and *KEAP1* were more significant than *RB1*. We focused on *RB1* here to pursue comparisons with SCLC.

**Table 2:** Multivariable Cox Proportional Hazards Model with backward selection procedure for NSCLC cohort

Factor/gene	Hazard ratio (95% CI)	*P*‐value
*MLL2* (mutant vs wild)	2.28 (1.43, 3.63)	0.001
Age (per year increase)	1.01 (0.99, 1.02)	0.637
Sex (female vs male)	1.06 (0.71, 1.58)	0.784
Stage (3 vs 4)	0.73 (0.44, 1.2)	0.217
Smoking (yes vs no)	1.42 (0.76, 2.66)	0.278
*KEAP1* (mutant vs wild)	2.8 (1.71, 4.59)	<0.001
*RB1* (mutant vs wild)	3.07 (1.54, 6.14)	0.002
*CRLF2* (mutant vs wild)	4.97 (1.12, 22.13)	0.036
*BRIP1* (mutant vs wild)	2.52 (1.28, 4.96)	0.007
*NFE2L2* (mutant vs wild)	3.51 (1.05, 11.69)	0.041
*ABL2* (mutant vs wild)	4.13 (1.21, 14.16)	0.024
*FAT1* (mutant vs wild)	0.37 (0.14, 0.93)	0.035
Chemotherapy (no vs yes)	2.73 (1.73, 4.29)	<0.001

The *RB1* wt NSCLC group had 12 patients with *EGFR* Exon 18‐21 alterations and 4 patients with *ALK* gene rearrangement. When we excluded these patients with targetable, driver mutations due to a more favorable outcome, *RB1* mutant NSCLC patients still had worse outcomes. There was one NSCLC patient with *EGFR* exon 19 deletion and *RB1* alteration in our cohort. The *RB1* alteration in this patient was found on repeat biopsy at the time of progression on first generation EGFR tyrosine kinase inhibitor (TKI), along with an acquired *EGFR* T790M mutation and a histopathology of adenocarcinoma. Transformation of *EGFR* mutant NSCLC to SCLC has been associated with loss of *RB1*.^20^ The *EGFR* exon 19 deletion at the time of initial diagnosis was found on a limited gene panel analysis. It is unclear if the *RB1* alteration represented a new event at the time of progression.

We looked at the association of *RB1* mutation with response to immunotherapy in NSCLC by analyzing our data of 97 NSCLC patients treated with immunotherapy with second line nivolumab or first line pembrolizumab. We had genomic data available on 66 of these patients. There were 6 patients with *RB1* alteration and none of these 6 patients responded to immunotherapy. In contrast, in those without *RB1* alteration (*N *=* *60), the response rate was 26.2%. The lack of response in all of the six patients with *RB1* mutation requires further evaluation in larger cohorts.

**Figure 2:**
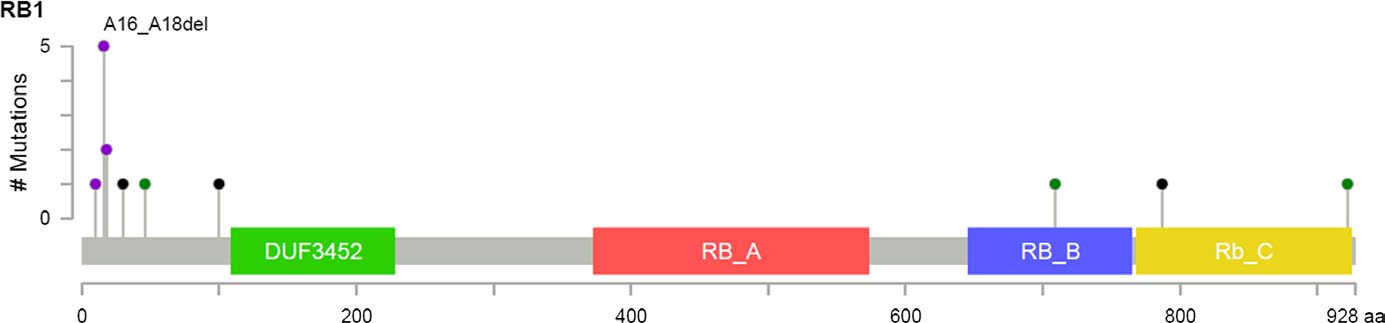
Mutation Distribution along RB Protein in NSCLC. Missense mutations labeled in green. Truncating mutations (nonsense, frameshift deletion, frameshift insertion, splice site) labeled in black. Single nucleotide polymorphisms (SNPs) and exon loss labeled in purple. DUF = Domain of unknown function (green). RB_A (red) and RB_B (blue) domains contain cyclin folds. RB_B also contains LXCXE binding site. RB_C (yellow) is the C terminal domain which binds E2F complexes

The distribution of mutations along the RB protein is depicted in Figure 2. The mutations in NSCLC were concentrated in the amino (N) terminal region. We identified a polyalanine deletion of amino acid 16‐18 in the N terminal region of RB which was seen in 5 of 16 patients with NSCLC (Table 3). This mutation has been described with an allelic frequency of 0.4% in ClinVar and predicted to be benign using the PolyPhen‐2 tool to predict the effect of amino acid alterations on protein structure. *RB1* mutation status was still predictive of poorer outcomes in NSCLC when we reanalyzed our data based upon mutations predicted to be benign (missense and deletions) or of unknown significance (splice site mutations) vs those predicted to be damaging (exon loss and nonsense mutations).

**Table 3:** Retinoblastoma (*RB1*) mutation type, protein alteration, RB IHC, and p16^INK4A^ scoring in NSCLC

Hugo_Symbol	Sample_ID	Protein_Change	Mean allelic frequency	Mutation_Type	RB IHC scoring, % IHC+ cells	P16^INK4A^ IHC scoring, % IHC+ cells
*RB1*	1	A16_A18del	0.47	Deletion	1+, 30%	0, 0
*RB1*	2	A16_A18del	0.41	Deletion	1+, 40%	0, 0
*RB1*	3	A16_A18del	0.45	Deletion	2+, 100%	2+, 20%
*RB1*	4	A16_A18del	0.38	Deletion	2+, 80%	0, 0
*RB1*	5	A16_A18del	0.59	Deletion	n/a	n/a
*RB1*	6	Y709C	0.26	Missense_Mutation	n/a	n/a
*RB1*	7	A18S	0.17	Missense_Mutation	n/a	n/a
*RB1*	8	R46T	0.22	Missense_Mutation	n/a	n/a
*RB1*	9	T922A	0.44	Missense_Mutation	n/a	n/a
*RB1*	10	Splice site 2326‐1G>T	0.71	Splice_Site	2+, 10%	2+, 100%
*RB1*	11	Splice site 1049+1G>T	0.47	Splice_Site	2+, 5%	2+, 100%
*RB1*	12	Loss, exons 18‐23		Loss	0, 0	2+, 100%
*RB1*	13	Loss exons 10‐11		Loss	0, 0	2+, 100%
*RB1*	14	G100*	n/a	Nonsense_Mutation	0, 0	2+, 100%
*RB1*	15	E30*	0.64	Nonsense_Mutation	n/a	n/a
*RB1*	16	R787*	n/a	Nonsense_Mutation	n/a	n/a

n/a, not available.

**Figure 3:**
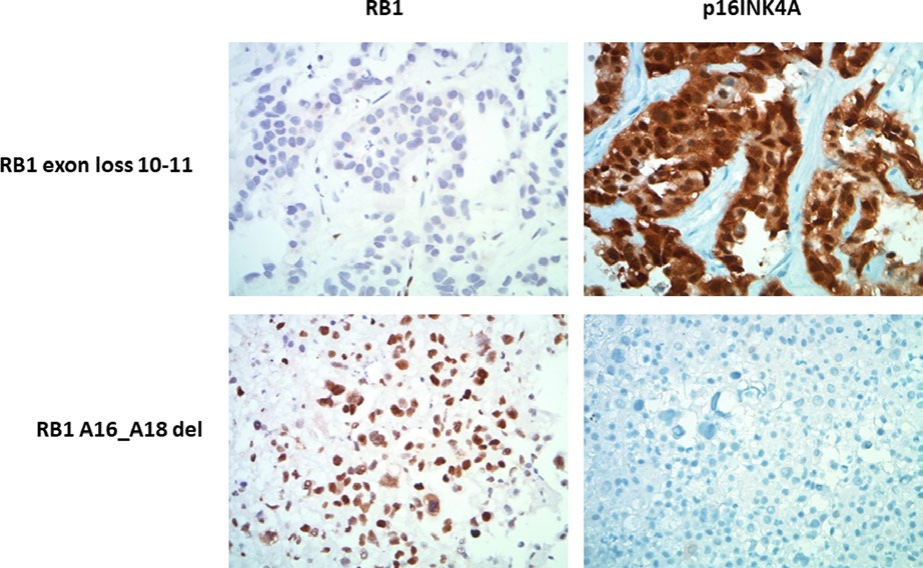
Immuno‐histochemical detection of RB1 and p16INK4A in two *RB1* mutant NSCLC tumors (400X). Description of exact *RB1* mutation given on left

We next looked at the expression of RB and the cyclin dependent kinase inhibitor p16^INK4A^ (CDKN2A) by IHC in relation to *RB1* mutation status in the NSCLC cohort (Figure 3 and Table 3). Nine of 16 patients had tumor tissue available for IHC. *RB1* exon loss and nonsense mutation were associated with a complete absence of RB expression in IHC and strongly intense p16^INK4A^ expression. *RB1*splice site alterations showed limited RB expression and strongly intense p16^INK4A^ expression. For four patients with polyalanine deletion and available tissue, RB expression was positive but variable and p16^INK4A^ IHC was negative. We had tissue on 9 *RB1* wt for IHC controls. *RB1* wt was associated universally with RB expression. p16^INK4A^ expression was variable in the *RB1* wt (data not shown).

We recorded the co‐mutations in the 16 *RB1* mutant NSCLC. *CDKN2A* damaging alterations, *CDK4* alteration, *CCND1* amplification and *CCNE1* amplification were seen in 3, 1, 1, and 2 patients, respectively. *TP53* mutations were present in 12 patients, followed by *STK11* mutations in 6 patients and *KRAS* mutations in four patients.

## 4 DISCUSSION

We found *RB1* mutant status to be strongly associated with worse outcomes in NSCLC. There have been prior studies looking at the clinical correlation of *RB1* in NSCLC with varying results, summarized in Table 4. The largest of these by Choi et al used DNA sequencing and identified *RB1* mutant status to be associated with shorter disease‐free survival only in early stage adenocarcinoma.^21^ The incidence of *RB1* mutation of 5.9% in their cohort is comparable to ours. Their cohort represented 49% never smokers, which might represent a different patient population than the average 10% never‐smokers with NSCLC.^22^ Geradts et al^23^ looked at RB expression through IHC in resected NSCLC and did not find any relation to outcome. Their results also showed an inverse relationship between p16^INK4A^ and RB expression. In a cohort of 73 patients with Stage I and II disease, Zhao et al^24^ reported RB expression by IHC to be associated with poorer outcomes. In another cohort of 106 patients, RB expression in resected NSCLC did not correlate with outcomes.^25^ *RB1* was altered in 7% of the lung adenocarcinoma TCGA 2014 dataset,^5^ but had no effect on survival (203 cases, 13 mutant *RB1, P *=* *0.994) (assessed via cBioPortal 10/2018). This TCGA dataset is most comparable to ours since our cohort was largely adenocarcinoma. A potential difference to explain our highly significant results is that our study focused on locally advanced and advanced NSCLC, as opposed to the early stage disease examined by all the other studies, including TCGA. Our study also used alterations at the DNA level as detected by targeted exome sequencing.

**Table 4:** Studies evaluating retinoblastoma (*RB1*) alteration in other NSCLC studies

Study	*N*	Stage	Histology	Technique	%RB positive	Clinical correlation
Geradts (1999)	103	Resected Stage I, II, III (*N *=* *58, 22, 23)	SCC (40) AdenoCa (44)	IHC	86.4%	NS
Jin (2001)	106	Resected Stage I, II	SCC (34) AdenoCa (72)	IHC	48%	NS
Zhao (2012)	73	Resected Stage I, II	Non Squamous	IHC	43.8%	RB+ poor
Choi (2015)	247	65% stage I,16% stage II & III, 2% stage IV, 49% never smokers	AdenoCa	WES CNV	*RB1* mutation 5.9%	*RB1* alteration shorter DFS (stage I, II)

NS, not significant; IHC, immunohistochemistry; SCC, squamous cell carcinoma; AdenoCa, adenocarcinoma; WES, whole exome sequencing; CNV, copy number variation; DFS, disease free survival.

The baseline characteristics of the *RB1* mutant NSCLC compared to *RB1* wt patients were well matched except for a significantly higher number of Stage III patients in the *RB1* mutant group. This imbalance had no effect on survival outcomes, however, because multivariate analysis showed the effect of stage on OS was not statistically significant (Table 2). This lack of statistical significance could be due to the low number of stage III patients in our cohort. An intriguing explanation would be that the recent (2014) addition of immunotherapy to stage IV patient care has increased their survival to 2‐3 years. The use of immunotherapy for stage III patients only started in 2018 and therefore is not reflected in this dataset. There is also a suggestion that the proportion of black race is greater in the *RB1* mutant group, which may bias survival outcomes. While race and *RB1* mutation status were indeed associated (*P *=* *0.011), the effect of *RB1* mutation status on survival was essentially unchanged after controlling for the effect of race in the Cox model, with race not significant in predicting survival (*P *=* *0.236).

We noted a concentration of *RB1* alterations to the N terminal domain in our NSCLC cohort. Regulation of the cell cycle by RB is primarily attributed to the conserved central pocket (amino acids 379‐792) and carboxy (C) terminal region (amino acids 792‐928). The crystal structure of the entire RB N terminal domain is not well understood but there is a suggestion that the N terminal domain is well conserved and interacts with the pocket domains.^26^ Because a significant number of missense mutations and exon deletions map to the N terminal domain in retinoblastoma patients, similar to our NSCLC cohort, it is possible that the mutations we observed affect an unknown critical function of RB.

Another reason we focused on advanced stage disease was to better compare the NSCLC results with our recently published genomic studies of small cell lung cancer (SCLC), where we found that mutant *RB1* status was associated with favorable survival outcomes.^8^ This remained true even in a more recent and expanded analysis examining 64 SCLC patients with largely extensive stage disease, in which *RB1* mutation was seen in 75% of SCLC cases and was associated with significantly better OS when compared with *RB1* wt status.^16^ The contrasting association of *RB1* mutation status with outcomes in NSCLC compared with SCLC is intriguing. The role of *RB1* in different malignancies and different contexts may be more complicated than simple regulation of cell cycle or response to chemotherapy.^27^ RB is inactivated by various mechanisms and there is a complex interplay between cyclin inhibitors and cyclin dependent kinases. It is possible that differences in co‐mutations may in part explain the differential outcomes that we saw in *RB1* mutated NSCLC compared to SCLC. Mutations in SCLC were distributed throughout the protein (Supporting Information, Figure S1A) and most of these were predicted to be damaging (Supporting Information, Figure S1B).

*RB1* mutation status and its relationship to outcomes has also been reported for breast cancer with contrasting results.^28^, ^29^ SCLC is a chemosensitive disease with initial response rates to chemotherapy for extensive stage SCLC of about 70%. *RB1* wt in SCLC was associated with a chemorefractory response (*P *=* *0.0334) and identifies a subset with poor outcomes.^16^ This indicates that loss of *RB1,* which has so far been considered a hallmark of SCLC, plays a role in making this disease initially chemosensitive. This is likely due to the absence of G1/S regulation in *RB1* mutated SCLC and accumulation of DNA adducts caused by platinum agents. Similarly, RB pathway disruption correlated with complete pathological response to neoadjuvant chemotherapy in a study on breast cancer.^29^

We also noted a lack of response to immunotherapy in *RB1* mutated NSCLC. Tumor genomics likely impacts the immune milleu of the tumors and potentially plays a role in response to immunotherapy. Patients with *EGFR* mutations and *ALK* gene rearrangements are known to have poor responses to immunotherapy and have been excluded from recent immunotherapy trials.^30^ PDL‐1 expression and tumor mutation burden^31^ are known biomarkers for predicting response to immunotherapy. High PDL‐1 expression predicts a response rate of about 50% to immunotherapy^30^ but depending on the cut off, patients with low PDL‐1 expression respond to immunotherapy as well, highlighting the imperfections of the currently available markers and the need to identify additional markers.

There is emerging data on the role of RB in mediating immune response in addition to its regulation of the cell cycle. In hepatocellular carcinoma, the retinoblastoma pathway has been proposed to regulate innate immune response. RB depletion in hepatoma cells resulted in a compromised immunological response to multiple stimuli.^32^ In bladder cancer, RB under expression was predictive of poor response to bacille Calmette‐Guerin (BCG) therapy in concert with interferon‐alpha (IFNα) therapy, providing further evidence that RB plays a role in mediating immune response.^33^, ^34^ In cervical cancer, human papilloma virus (HPV) oncoprotein E7 is known to bind to RB and cause its inactivation. DNA vaccine where E7 was altered to evade RB binding was more immunogenic compared to unaltered E7, further establishing the role of RB in immune response.^35^ Our single institution dataset indicates *RB1* mutation to be associated with a lack of response to immunotherapy in NSCLC. Coupled with these observations in other malignancies and biological explanations for the role of RB in mediating immune response, the association of *RB1* mutation with lack of response to immunotherapy should be evaluated in larger cohorts of NSCLC.

This is a single institution review. Patients who underwent next generation sequencing may represent a specific cohort with access to different treatments. We did not have paired germ line sequencing^36^ for these patients to ascertain whether all of these alterations were somatic in nature. Germ line testing at this time is not considered standard of care. We recognize that Stage III lung cancer in itself represents a disease with varying outcomes. Our cohort represents a mixed population of stage III, stage IV, and recurrent NSCLC, but there is general consensus about treatment within a single institution. The majority of our NSCLC patients displayed adenocarcinoma histology, reflecting the practice pattern of genomic sequencing in these patients.

## CONFLICT OF INTEREST

None declared.

## Supporting information

* Click here for additional data file.
