# Supplementary material for: Retinoblastoma mutation predicts poor outcomes in advanced non small cell lung cancer
Source: Cancer Med. 2019 Feb 17;8(4):1459–66. doi: 10.1002/cam4.2023 (PMC6488103; doi:10.1002/cam4.2023)

Supplement Table 1: DNA Gene List: Entire Coding Sequence for the Detection of Base Substitutions, Insertion/Deletions, and Copy Number Alterations

ABL1 ABL2 ACVR1B AKT1 AKT2 AKT3 ALK AMER1 (FAM123B) APC AR ARAF ARFRP1 ARID1A ARID1B ARID2 ASXL1 ATM ATR ATRX AURKA AURKB AXIN1 AXL BAP1 BARD1 BCL2 BCL2L1 BCL2L2 BCL6 BCOR BCORL1 BLM BRAF BRCA1 BRCA2 BRD4 BRIP1 BTG1 BTK C11orf30 (EMSY) CARD11 CBFB CBL CCND1 CCND2 CCND3 CCNE1 CD274 CD79A CD79B CDC73 CDH1 CDK12 CDK4 CDK6 CDK8 CDKN1A CDKN1B CDKN2A CDKN2B CDKN2C CEBPA CHD2 CHD4 CHEK1 CHEK2 CIC CREBBP CRKL CRLF2 CSF1R CTCF CTNNA1 CTNNB1 CUL3 CYLD DAXX DDR2 DICER1 DNMT3A DOT1L EGFR EP300 EPHA3 EPHA5 EPHA7 EPHB1 ERBB2 ERBB3 ERBB4 ERG ERRFI1 ESR1 EZH2 FAM46C FANCA FANCC FANCD2 FANCE FANCF FANCG FANCL FAS FAT1 FBXW7 FGF10 FGF14 FGF19 FGF23 FGF3 FGF4 FGF6 FGFR1 FGFR2 FGFR3 FGFR4 FH FLCN FLT1 FLT3 FLT4 FOXL2 FOXP1 FRS2 FUBP1 GABRA6 GATA1 GATA2 GATA3 GATA4 GATA6 GID4 (C17orf39) GLI1 GNA11 GNA13 GNAQ GNAS GPR124 GRIN2A GRM3 GSK3B H3F3A HGF HNF1A HRAS HSD3B1 HSP90AA1 IDH1 IDH2 IGF1R IGF2 IKBKE IKZF1 IL7R INHBA INPP4B IRF2 IRF4 IRS2 JAK1 JAK2 JAK3 JUN KAT6A (MYST3) KDM5A KDM5C KDM6A KDR KEAP1 KEL KIT KLHL6 KMT2A (MLL) KMT2C (MLL3) KMT2D (MLL2) KRAS LMO1 LRP1B LYN LZTR1 MAGI2 MAP2K1 MAP2K2 MAP2K4 MAP3K1 MCL1 MDM2 MDM4 MED12 MEF2B MEN1 MET MITF MLH1 MPL MRE11A MSH2 MSH6 MTOR MUTYH MYC MYCL (MYCL1) MYCN MYD88 NF1 NF2 NFE2L2 NFKBIA NKX2-1 NOTCH1 NOTCH2 NOTCH3 NPM1 NRAS NSD1 NTRK1 NTRK2 NTRK3 NUP93 PAK3 PALB2 PARK2 PAX5 PBRM1 PDCD1LG2 PDGFRA PDGFRB PDK1 PIK3C2B PIK3CA PIK3CB PIK3CG PIK3R1 PIK3R2 PLCG2 PMS2 POLD1 POLE PPP2R1A PRDM1 PREX2 PRKAR1A PRKCI PRKDC PRSS8 PTCH1 PTEN PTPN11 QKI RAC1 RAD50 RAD51 RAF1 RANBP2 RARA RB1 RBM10 RET RICTOR RNF43 ROS1 RPTOR RUNX1 RUNX1T1 SDHA SDHB SDHC SDHD SETD2 SF3B1 SLIT2 SMAD2 SMAD3 SMAD4 SMARCA4 SMARCB1 SMO SNCAIP SOCS1 SOX10 SOX2 SOX9 SPEN SPOP SPTA1 SRC STAG2 STAT3 STAT4 STK11 SUFU SYK TAF1 TBX3 TERC TERT(promoter only) TET2 TGFBR2 TNFAIP3 TNFRSF14 TOP1 TOP2A TP53 TSC1 TSC2 TSHR U2AF1 VEGFA VHL WISP3 WT1 XPO1 ZBTB2 ZNF217 ZNF703

DNA Gene List: For the Detection of Select Rearrangements

ALK BCL2 BCR BRAF BRCA1 BRCA2 BRD4 EGFR ETV1 ETV4 ETV5 ETV6 FGFR1 FGFR2 FGFR3 KIT MSH2 MYB MYC NOTCH2 NTRK1 NTRK2 PDGFRA RAF1 RARA RET ROS1 TMPRSS2

Supplement Figure 1:

A. Mutation Distribution along RB Protein in SCLC.

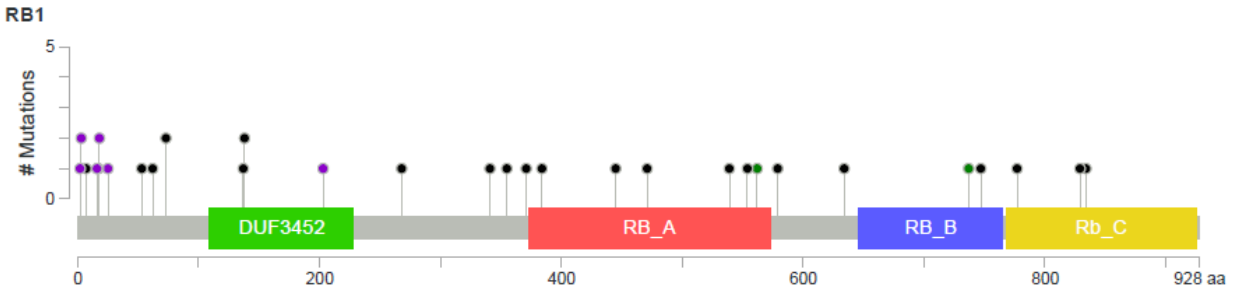

Missense mutations labeled in green. Truncating mutations: nonsense, frameshift deletion, frameshift insertion, and splice site labeled in black. SNPs and exon loss in purple. DUF= Domain of unknown function.

B. Comparison of RB1 mutation distribution in NSCLC vs SCLC.

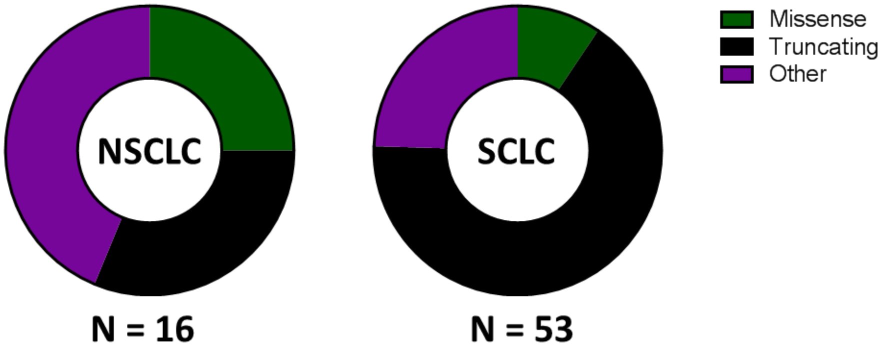

Supplement: Supplementary file 1 — * [file CAM4-8-1459-s001.pdf]
